# CD26 Inhibition Potentiates the Therapeutic Effects of Human Umbilical Cord Blood-Derived Mesenchymal Stem Cells by Delaying Cellular Senescence

**DOI:** 10.3389/fcell.2021.803645

**Published:** 2022-02-01

**Authors:** Miyeon Kim, Jinyoung Go, Ji Hye Kwon, Hye Jin Jin, Yun Kyung Bae, Eun-Young Kim, Eun-Ju Chang, Soo Jin Choi, Seong Who Kim

**Affiliations:** ^1^ Biomedical Research Institute, MEDIPOST Co., Ltd., Seongnam, South Korea; ^2^ Department of Biochemistry and Molecular Biology, Asan Medical Center, University of Ulsan College of Medicine, Seoul, South Korea; ^3^ Department of Biomedical Sciences, Asan Medical Center, University of Ulsan College of Medicine, Seoul, South Korea

**Keywords:** CD26, DPP4, mesenchymal stem cell, cellular senescence, emphysema

## Abstract

Mesenchymal stem cells (MSCs) are recognized as potential treatments for multiple degenerative and inflammatory disorders as a number of animal and human studies have indicated their therapeutic effects. There are also several clinically approved medicinal products that are manufactured using these cells. For such large-scale manufacturing requirements, the *in vitro* expansion of harvested MSCs is essential. Multiple subculturing of MSCs, however, provokes cellular senescence processes which is known to deteriorate the therapeutic efficacy of the cells. Strategies to rejuvenate or selectively remove senescent MSCs are therefore highly desirable for fostering future clinical applications of these cells. In this present study, we investigated gene expression changes related to cellular senescence of MSCs derived from umbilical cord blood and found that CD26, also known as DPP4, is significantly upregulated upon cellular aging. We further observed that the inhibition of CD26 by genetic or pharmacologic means delayed the cellular aging of MSCs with their multiple passaging in culture. Moreover, the sorting and exclusion of CD26-positive MSCs from heterogenous cell population enhanced *in vitro* cell attachment and reduced senescence-associated cytokine secretion. CD26-negative MSCs also showed superior therapeutic efficacy in mouse lung emphysema model. Our present results collectively suggest CD26 is a potential novel target for the rejuvenation of senescent MSCs for their use in manufacturing MSC-based applications.

## Introduction

Since mesenchymal stem cells (MSCs) were identified as colony-forming cells in addition to hematopoietic stem cells in the bone marrow (Friedenstein et al., 1968), they have attracted the interest of many researchers due to their ability to differentiate into multiple lineages with undemanding culture condition. The multipotential differentiation capacity of MSCs led its potential clinical application to tissue repair and regeneration. However, once it was suggested MSCs could be used to relieve adverse immune reaction like graft versus host disease (GVHD) after a bone marrow transplantation (Lazarus et al., 2005; Le Blanc et al., 2008), their role as paracrine effector and immunomodulator emerged. Subsequent studies have now revealed that MSCs regulate immune response by secreting anti-inflammatory cytokines such as prostaglandin E2 and transforming growth factor β (Aggarwal and Pittenger, 2005; English et al., 2009). Furthermore, induced changes in the composition of T lymphocytes by MSCs toward an increased regulatory T cells or anti-inflammatory type 2 helper T cells has also been observed (Prevosto et al., 2007; Bai et al., 2009). The first drug to be produced using cryopreserved allogeneic adult bone marrow MSCs, Prochymal, was approved by several countries for the treatment of refractory acute GVHD in 2012, and there have now been over 1,000 clinical trials that are registered at Clinicaltrials.gov to treat conditions ranging from knee osteoarthritis to Crohn’s disease.

Regardless of promising results from preclinical and clinical studies of the simple administration of naïve MSCs, conflicting outcomes regarding their clinical benefits have been reported in some clinical trials (Herreros et al., 2012; Weiss et al., 2013) necessitating the need for a more developed understanding of molecular and cellular mechanisms that affect clinical efficacy of these stem cells. As therapeutic effectiveness of MSCs can be influenced by a number of factors such as the donor, harvest site, and how they are expanded *in vitro* (Choudhery et al., 2014; Heo et al., 2016; Nikolits et al., 2021), there have been efforts to strengthen or fine-tune their therapeutic effects for certain diseases through physical, chemical, or genetic modulation. A recent study reported that ectopic expression of Nanog could restore myogenic differentiation potential of MSCs (Han et al., 2012). Another group of researchers demonstrated that hypoxia-priming can be used to achieve better clinical outcome from the use of MSCs to treat diseases such as GVHD (Kim et al., 2018). In this regard, we have focused our own research attention on identifying target molecules that can be manipulated to enhance the therapeutic efficacy of MSCs, especially those factors related to senescence. Cellular senescence is a phenomenon in which division cycle of the cell is permanently halted. The senescence eventually arisen from the *in vitro* expansion of MSCs impedes efficient production of drugs by these cells. Furthermore, senescent MSCs have been shown to no longer exhibit immunosuppressive properties, such as attenuation of T lymphocyte proliferation (Chinnadurai et al., 2017) and secrete higher level of pro-inflammatory cytokines than their pre-senescent counterparts (Sepúlveda et al., 2014). Strategies to slow down or reverse the process of MSC senescence would therefore be beneficial in terms of both the yield and efficacy of these cells for medical applications.

Here, we demonstrate that CD26, also known as DPP4, is senescence-related protein showing increased expression with passaging in MSCs. We also show that pharmacologic inhibition or knockdown of CD26 prevent the progression of senescence-related phenotype in MSCs. Further analysis has revealed CD26-negative MSCs exhibited improved migratory and secretory phenotype. Finally, we show that CD26-depleted MSCs exhibited superior engraftment and therapeutic efficacy when used to treat tissue damage in a mouse emphysema model.

## Materials and Methods

### Cell Culture

The Institutional Review Board of MEDIPOST Co., Ltd. approved this study (MP-2015-6-5). Human umbilical cord blood (hUCB) was obtained from full-term deliveries with maternal informed consent. To isolate human umbilical cord blood-derived mononuclear cells (hUCB-MNCs), hUCB samples were fractionated using a Ficoll-Hypaque solution (density = 1.077 g/cm^3^; GE Healthcare, Uppsala, Sweden) density gradient centrifuge at 400 g for 30 min. Cells were cultured with a density of 5 × 10^4^ cells/cm^2^ in α-minimum essential medium (α-MEM; Gibco. Calsbad, CA, United States) supplemented with 10% (v/v) fetal bovine serum (FBS; Gibco). Cell surface marker expression profile and representative tri-lineage differentiation images are shown in Supplementary Figure S1. Cells were maintained at 37°C in a humidified atmosphere with 5% CO_2_ and Culture medium was replaced twice a week. Cells were subcultured at 50% confluence. Cumulative population doubling (CPD) was calculated for each passage based on the total number of cells. Number of cell doublings was calculated by dividing the number of harvested cells by the number of seeded cells.

### Measurement of Cell Area and Size

To measure the cell area, images were acquired at 100X magnification by a light microscopy equipped with an ECLIPSE TS100 (Nikon Instruments Inc., Melville, NY, United States). ImageJ software (National Institutes of Health, Bethesda, MD, United States) was used to draw cell margins and measure cell area. To evaluate cell size, trypsinized cells were imaged at 100X magnification. The diameter and circularity of cells were measured by Image-Pro Plus 7 software (Media Cybernetics, Rockville, MD, United States).

### Senescence-Associated Beta-Galactosidase Staining

A Senescence β-Galactosidase Staining Kit (Cell Signaling Technology, Danvers, MA, United States) was used in accordance with the manufacturer’s instruction. Cells that appeared with blue stain under a bright-field microscope were counted as positive for SA β-gal.

### Immunoblotting

Cells were lysed in RIPA buffer (ThermoFisher scientific, Waltham, MA, United States) with ultrasonication (Branson, Merck, Burlington, MA, United States). Cellular extracts were separated by SDS-PAGE on BoltTM bis-tris plus gel (Invitrogen, Calsbad, CA, United States). Antibodies raised against the following proteins were used for signal detection: p53, p21, p16 (Cell Signaling Technology), pRb, vitronectin (Abcam, Cambridge, United Kingdom), integrin αⅤβ3 (Novus biologicals, Centennial, CO, United States), and β-actin (Sigma-Aldrich, St Louis, MO, United States).

### Flow Cytometry and Cell Sorting

Cells were stained for 10 min at room temperature with the antibody against specific protein or mouse IgG2a isotype antibody. The cells were then washed with Dulbecco’s phosphate buffered saline (D-PBS; Corning, Manassas, VA, United States) and fixed with 1% paraformaldehyde. Protein expression was measured by flow cytometry on a MACSQuant Analyzer 10 (Miltenyi Biotec GmbH). At least 10,000 gated cells were used for the measurement of expression. Antibodies against the following proteins were used for flow cytometry analysis: CD26-PE (Miltenyi Biotec GmbH, Bergisch Gladbach, Germany), CD34-FITC, CD45-FITC, CD73-PE (BD Bioscience, San Diego, CA, United States), CD90-PE, CD105-PE (eBioscience, San Diego, CA, United States). To isolate cells based on their expression level of CD26, cells were incubated with CD26 antibody for 10 min at 4°C. Cells with a CD26 expression level above 85th percentile were sorted as CD26-positive (CD26^+^) and below 15th percentile were sorted as CD26-negative (CD26^−^). A FACSAria II (BD Bioscience) was used for cell sorting.

### Animal Model

All animal experiments were reviewed and approved by the Institutional Animal Care and Use Committee of MEDIPOST Co., Ltd. (MP-LAR-2017-12-1). Six-week-old C57BL/6 mice were purchased from Samtako bio Korea CO., Ltd. (Osan-si, Gyeonggi-do, Korea). These mice were allowed to acclimate for 1 week prior to conducting the experiments and were divided randomly into four groups (*n* = 10 in each) as follows: (A) a naïve group, (B) elastase group, (C) elastase with CD26^+^ hUCB-MSCs (passage 6) group, (D) elastase with CD26^−^ hUCB-MSCs (passage 6) group. To produce elastase-induced emphysema, all mice except those in the group A were intratracheally instilled with 0.4 U porcine pancreatic elastase (Sigma-Aldrich). The mice in group A were instead treated with intratracheal instillation of D-PBS. The mice in groups C and D were injected intravenously with 2 × 10^4^ UCB-MSCs 1 week after elastase treatment. All mice were sacrificed on 14 days after elastase treatment.

### Histologic Analysis of the Mouse Lung

Harvested lungs form the mice were perfused with PBS and inflated *via* an intratracheal infusion of 0.5% low-melting agar. The lungs were then fixed with 10% formaldehyde solution for 24 h and processed for paraffin embedding, from which 4 μm-thick sections were made. A portion of these sections were stained with hematoxylin and eosin (H&E), and others were subjected to immunostaining. The average interalveolar distance was evaluated by measuring the mean linear intercept, as calculated previously by Cooney and Thurlbeck (Cooney and Thurlbeck, 1982). Tissue samples were stained with anti-human β2-microglobulin antibody (Santa Cruz biotechnology, Santa Cruz, CA, United States) to evaluate the engraftment of human cells in the lung. Nuclei were counterstained with Hoechst 33342 (Invitrogen, Grand Island, NY, United States). Fluorescent images were acquired and analyzed using an LSM 800 confocal microscope (Zeiss, Oberkochen, Germany).

### mRNA Isolation and Quantitative Reverse-Transcription PCR

RNA was extracted from cell pellets using TRIzol (Invitrogen). cDNA was then synthesized by superscript II reverse transcriptase (Invitrogen) and analyzed by qPCR using a LighCyclerTM 480 (Roche, Mannheim, Germany). Relative amount of cDNA was calculated by comparative cycle threshold (2-ΔΔCt) method with normalization to GAPDH cDNA. The sequences of the primers used are listed in Supplementary Table S1.

### RNA Interference

CD26 and control small interfering RNA (siRNA) were purchased from Dharmacon (Chicago, IL, United States). Pooled siRNA consisting of four duplexes were used in the experiments. Cells at passage 7 were transfected with siRNA using DharmaFECT (Dharmacon) in accordance with the manufacturer’s instructions. The sequences of the siRNA used are listed in Supplementary Table S1.

### Evaluation of Multilineage Differentiation Potential

Multilineage differentiation potential was evaluated by using specific culture conditions to induce differentiation into osteocytes or adipocytes as described previously (Jin et al., 2010). Von Kossa staining and ALP staining were performed to visualize the differentiation into osteocytes or osteoblasts. Safranin O staining was performed to visualize proteoglycan contents made by chondrocytes. Oil red O staining was performed to visualize lipid vacuoles in adipocytes. All staining procedures were performed in accordance with manufacturer’s instructions (Sigma-Aldrich). Images of the stained cells were quantified by calculating the ratio of stained cells to the total cell number.

### Cell Adhesion Assay

Cell adhesion was evaluated using IncuCyte (Essen Bioscience, Ann Arbor, MI, United States). Cells were seeded in six well culture dishes at 2,000 cells/cm^2^ and grown at 37°C in a 5% CO_2_ incubator in triplicate. Images of the cells were acquired after 48 h using automated image acquisition software. The numbers of attached cells were measured using the cell counter plugin in ImageJ software (National Institutes of Health).

### Extracellular Matrix Cell Adhesion Array

A Colorimetric ECM Cell Adhesion Array Kit (Merck) was used to estimate the extent of cell adhesion to various ECM components. Cells were seeded into 96 well culture plates coated with rehydrate ECM strips at 60,000 cells/well. The optical densities at 555 nm were then measured after 2 h using VersaMax Microplate Reader (Molecular Devices, Sunnyvale, CA, United States).

### Microarray Analysis

The microarray experiments were conducted by DNA Link Corporation (Seoul, Korea). Briefly, two replicates of RNA extracted from early (passage 4) or late (passage 10) hUCB-MSCs from same donor were analyzed using Affymetrix Human gene 2.0 Array Chip X. The generated microarray data files were deposited in GEO dataset (GSE183995).

### Differential Expressed Gene Identification and Gene Ontology Analysis

Raw data files (.CEL file) were read and analyzed by R 4.1.0 software, following the previously described Bioconductor workflow (Bernd Klaus, 2021). We used the limma 3.48.1 package (Ritchie et al., 2015) for identification of DEGs. Probes with fold changes >2 and an adjusted *p* value <0.05 were considered to be significant and included in the gene ontology analysis. the topGO 2.44.0 package (Adrian Alexa, 2021) was used for gene ontology enrichment analysis.

### Multiple Cytokine Analysis

After cell sorting was conducted based on CD26 expression as described above, the cells were seeded into six well plates at 100,000 cells/well. Conditioned media were harvested after 24 h and stored at −80°C. A Magnetic Luminex Screening Assay Human Premixed Multi-Analyte Kit (#LXSAHM-28, R&D Systems) was used to analyzed multiple cytokines simultaneously. Samples were processed by KOMA BIOTECH Inc. (Seoul, Korea) in accordance with the manufacturer’s instructions.

### Statistical Analysis

Data are presented as mean value ± standard deviation. Differences between two groups were analyzed using two-tailed *t*-test. Differences among groups of three or more were analyzed by one way ANOVA with Fisher’s LSD post-hoc test of variance via Prism six software (GraphPad, LA Jolla, CA, United States). *p* values less than 0.05 were considered statistically significant.

## Results

### CD26 Expression Increases in Senescent MSCs

**FIGURE 1:**
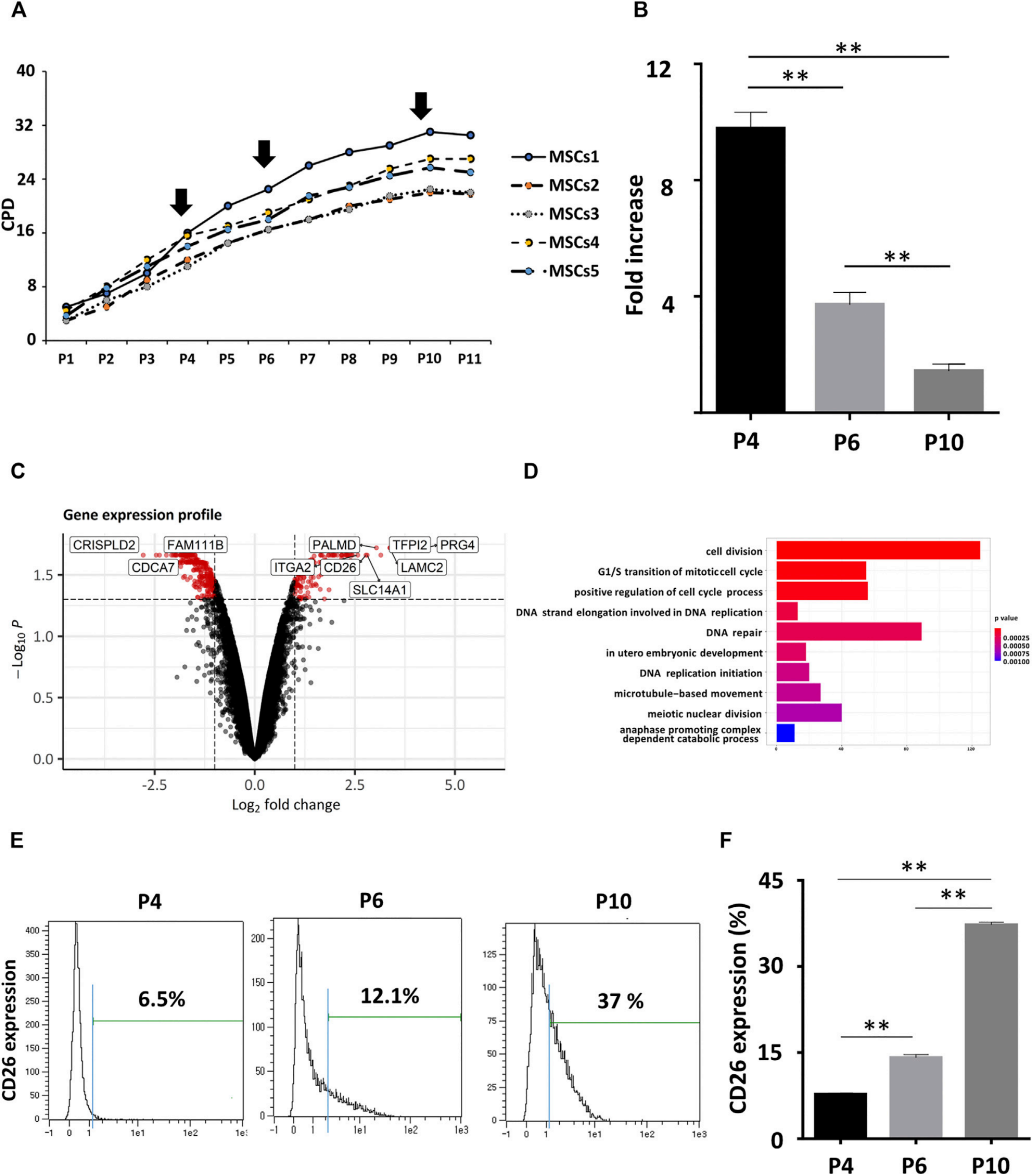
CD26 gene upregulation in aging hUCB-MSCs. **(A)** CPDs of hUCB-MSCs from five different donors. **(B)** Fold increase of hUCB-MSCs at each passage (*n* = 3). The fold increase was calculated by dividing the number of harvested cells by the number of seeded cells. **(C)** Volcano plot showing DEGs between early (passage 4) and late (passage 10) hUCB-MSCs. Genes with log2 fold change >1 and adjusted *p* value <0.05 are marked as red. **(D)** Bar graph of enriched GO terms from the DEGs in **(C)**. The X-axis represents the number of genes included for each specific GO terms. **(E)** CD26 expression in hUCB-MSCs at each passage measured by flow cytometry. An isotype control was used to determine the threshold level (blue line). **(F)** Bar graph showing ratio of CD26 expressing cells in **(D)** (*n* = 3). Abbreviation: hUCB-MSCs, human umbilical cord-derived blood mesenchymal stem cells; CPD, cumulative population doubling; DEG, differentially expressed gene; GO, gene ontology.

To gain new insights into the aging profile of MSCs, we measured cumulative population doubling (CPD) of five different human mesenchymal stem cell lines from umbilical cord blood (hUCB-MSCs; Figure 1A). The doubling rate of all five cell lines declined gradually and this decrease in the proliferation rate was significant among early (Passage 4, P4), intermediate (Passage 8, P8), and late (Passage 10, P10) passage cells (Figure 1B). The typical characteristics of senescent cells were evident in the late passages, such as increase in the cell area and size, and in the fraction of cells that were stained positively with senescence-associated β galactosidase (SA β-gal; Supplementary Figure S2A). Decreases in the expression of genes related to cell stemness as Oct4 and Nanog were observed by qPCR (Supplementary Figure S2B). Moreover, immunoblotting of lysates from the late passage hUCB-MSCs revealed prominent changes in the expression of proteins known for their association with cellular senescence, such as an accumulation of phosphorylated p53 (pho-p53), p21, and p16 and a decrease of phosphorylated Rb (pho-Rb) (Supplementary Figure S2C).

The differentiation potential of the hUCB-MSCs was assessed by Von Kossa or oil red O staining after they had been induced to differentiate into osteocytes or adipocytes. A significant decrease was found in the ratio of stained cells at late passages, suggesting a reduced stemness in senescent cells (Supplementary Figure S2D). These results indicated that well-known phenotypes related to senescence were reproduced in hUCB-MSCs. We then performed gene expression profiling with Affymetrix Human gene 2.0 Array chips to identify senescence-related differentially expressed genes (DEGs) in hUCB-MSCs. Two replicates of early (passage 4) or late (passage 10) hUCB-MSCs from same donor were used for microarray analysis. We detected 141 upregulated and 255 downregulated genes in senescent hUCB-MSCs (Figure 1C; Supplementary Table S2). We additionally performed gene ontology (GO) enrichment analysis to determine substantially affected pathways upon the onset of senescence in the MSCs. As expected, cell cycle and checkpoint related GO terms were most enriched in the lists of DEGs (Figure 1D). DNA repair was also among the enriched GO terms, possibly indicating the importance of DNA damage and repair processes in cellular senescence (d’Adda di Fagagna, 2008). Among the DEGs we identified, CD26 (DPP4, Dipeptidyl peptidase 4), membrane-bound protease, attracted our attention in particular as there is little current evidence to suggest causal link between CD26 and cell cycle or cellular aging. Also taken into consideration was that pharmacologic CD26 inhibitors are already available in the market for treatment of diabetes. The differential expression of CD26 was confirmed by flow cytometry. There was an observed rise in the proportion of cells expressing CD26 in late passage (Figures 1E,F). similar results were obtained using MSCs from umbilical cord tissue (Supplementary Figure S2E). In summary, we have identified significant DEGs in hUCB-MSCs, and confirmed the membrane-bound protease CD26 as one of upregulated genes in senescent hUCB-MSCs.

### The Suppression of CD26 Delays Cellular Aging in hUCB-MSCs

**FIGURE 2:**
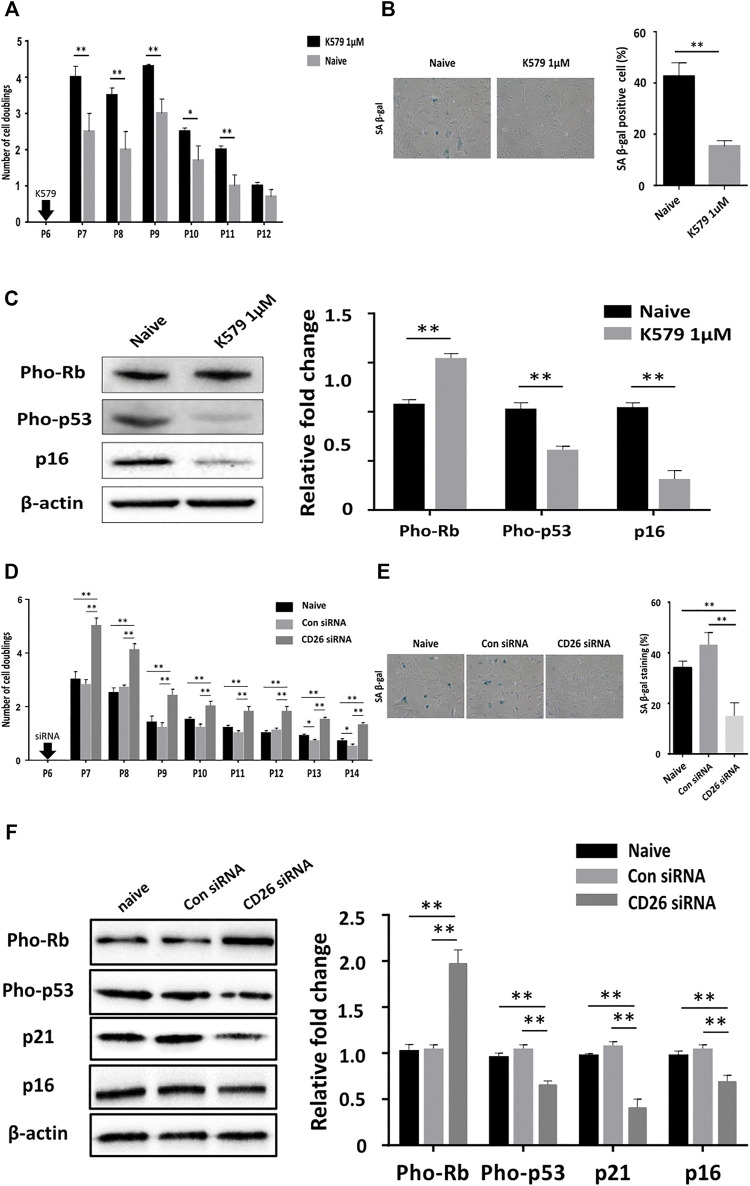
Pharmacologic inhibition of CD26 attenuates cellular senescence of hUCB-MSCs. **(A)** Number of cell doublings of hUCB-MSCs after K579 treatment (*n* = 3). **(B)** Representative Images **(left)** and bar graph **(right)** of SA β-gal stained naïve and K579-treated cells (*n* = 4, passage 10); Scale bar, 50 μm. **(C)** Immunoblotting images **(left)** and densitometry **(right)** of senescence-associated proteins in naïve and K579-treated cells (*n* = 3). **(D)** Number of cell doublings of hUCB-MSCs after transfection with scrambled control or CD26 siRNA (*n* = 3). **(E)** Representative images **(left)** and bar graph **(right)** of SA β-gal-stained cells of scrambled control- or CD26 siRNA- transfected hUCB-MSCs (*n* = 3, passage 10); Scale bar, 50 μm. **(F)** Immunoblotting images **(left)** and densitometry **(right)** of senescence-associated proteins in scrambled control- or CD26 siRNA-transfected hUCB-MSCs (*n* = 3, passage 6). All microscopic images were taken with 100X original magnification. Abbreviation: hUCB-MSCs, human umbilical cord blood mesenchymal stem cells; siRNA, small interfering RNA; SA β-gal, senescence-associated β galactosidase. *p < 0.05; **p < 0.01.

It was uncertain from our earlier results whether CD26 played any role in accelerating cellular senescence in hUCB-MSCs. To identify whether inhibition of CD26 can affect the progression of cellular aging, hUCB-MSCs were treated with K579, a slow acting CD26 inhibitor, at passage 6. The proliferation rate was found to be constantly higher in K579-treated cells and remain significant until passage 11 (Figure 2A). The proportion of cells stained with SA β-gal was also significantly lower in K579-treated cells (Figure 2B), suggesting the difference in cell proliferation with K579 treatment was due to a delay in senescence. Immunoblotting analysis confirmed the suppression of the senescence pathway as the pho-Rb levels increased, and those of pho-p53 and p16 decreased after K579 treatment (Figure 2C). Similar results were acquired when CD26 siRNA was used instead of K579 (Figures 2D–F). Of note, CD26 siRNA induced prolonged suppression of CD26 level over several passages (Supplementary Figure S2), which was consistent with the continuously increased proliferation rate observed upon CD26 inhibition. These results suggest that CD26 actively influences cellular proliferation by inducing cellular aging, and that the inhibition of CD26 can slow the progression of senescence in MSCs.

### The Surface Adhesion of hUCB-MSCs is Enhanced by the Inhibition of CD26

**FIGURE 3:**
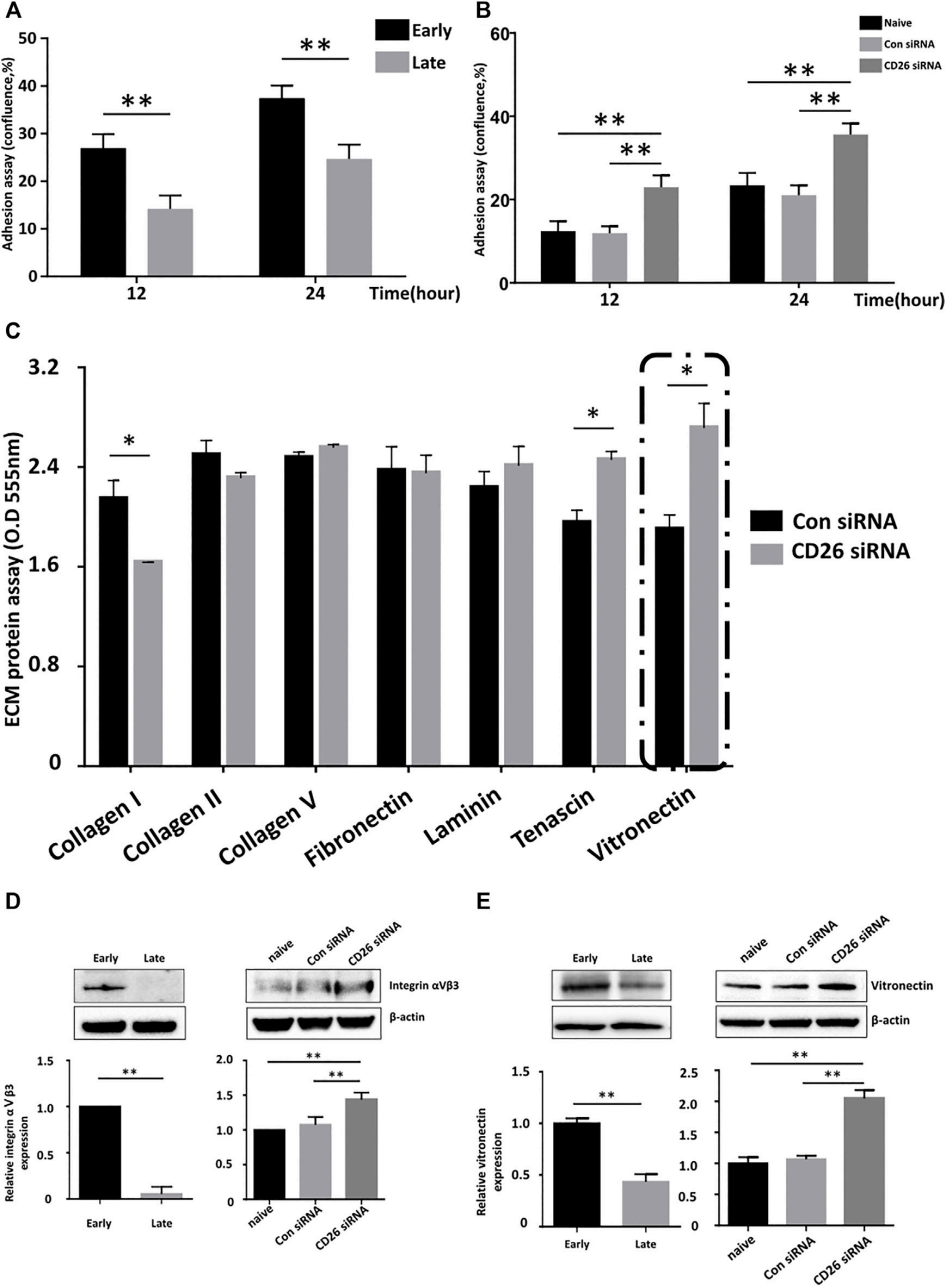
Knockdown of CD26 in hUCB-MSCs improves their attachment to the extracellular matrix. **(A)** Time-dependent changes in the confluence of early (passage 4) and late (passage 10) passage hUCB-MSCs (*n* = 3). **(B)** Time-dependent change in the confluence of scrambled control- or CD26 siRNA-treated hUCB-MSCs (*n* = 3). **(C)** Extracellular matrix cell adhesion array analyses of scrambled control- or CD26 siRNA-treated hUCB-MSCs (*n* = 3). **(D, E)** Immunoblotting images and densitometry of Integrin αⅤβ3 **(D)** and vitronectin **(E)** in early (passage 4) and late (passage 10) hUCB-MSCs **(left)** or hUCB-MSCs transfected with scrambled control or CD26 siRNA **(right)** (*n* = 3, passage 6). Abbreviation: hUCB-MSCs, human umbilical cord blood mesenchymal stem cells; siRNA, small interfering RNA. *p < 0.05; **p < 0.01.

Poor engraftment of administered MSCs into target tissues is regarded as one of the reasons for the limited duration of their clinical effects after injection (Zhao et al., 2014). We experimented whether inhibition of CD26 would improve MSC engraftment. A cell adhesion assay with hUCB-MSCs in early and late passage indicated greater attachment of viable MSCs at early passage (Figure 3A), showing cellular aging influences adhesion of cells to surface. Importantly, transfection of CD26 siRNA improves adhesion of MSCs (Figure 3B). We next attempted to identify an extracellular matrix (ECM) component that is affected by expression of CD26. We evaluated adhesion of MSCs to various ECM components using ECM cell adhesion array kit. The attachment of these cells to tenascin and vitronectin was significantly enhanced by the knockdown of CD26 (Figure 3C). As the adhesion to vitronectin was the most dramatically improved by suppression of CD26, we further investigated the expression of one of its ligands, integrin αVβ3. The integrin αVβ3 level was higher in early passage hUCB-MSCs than late passage hUCB-MSCs (Figure 3D, left), and knockdown of CD26 increased its expression (Figure 3D, right). Interestingly, CD26 siRNA also induced an increase in vitronectin expression (Figure 3E), suggesting CD26 is involved in the control of expression of both vitronectin and integrin αVβ3. Collectively, our results demonstrated that the inhibition of CD26 enhances ECM adhesion by MSCs, and that the upregulation of integrin αVβ3 and vitronectin may be among the mechanisms underlying this effect.

### Subpopulation of Cells With Highly Expressing CD26 Shows Premature Cellular Aging Leading to Pro-Inflammatory Secretome

**FIGURE 4:**
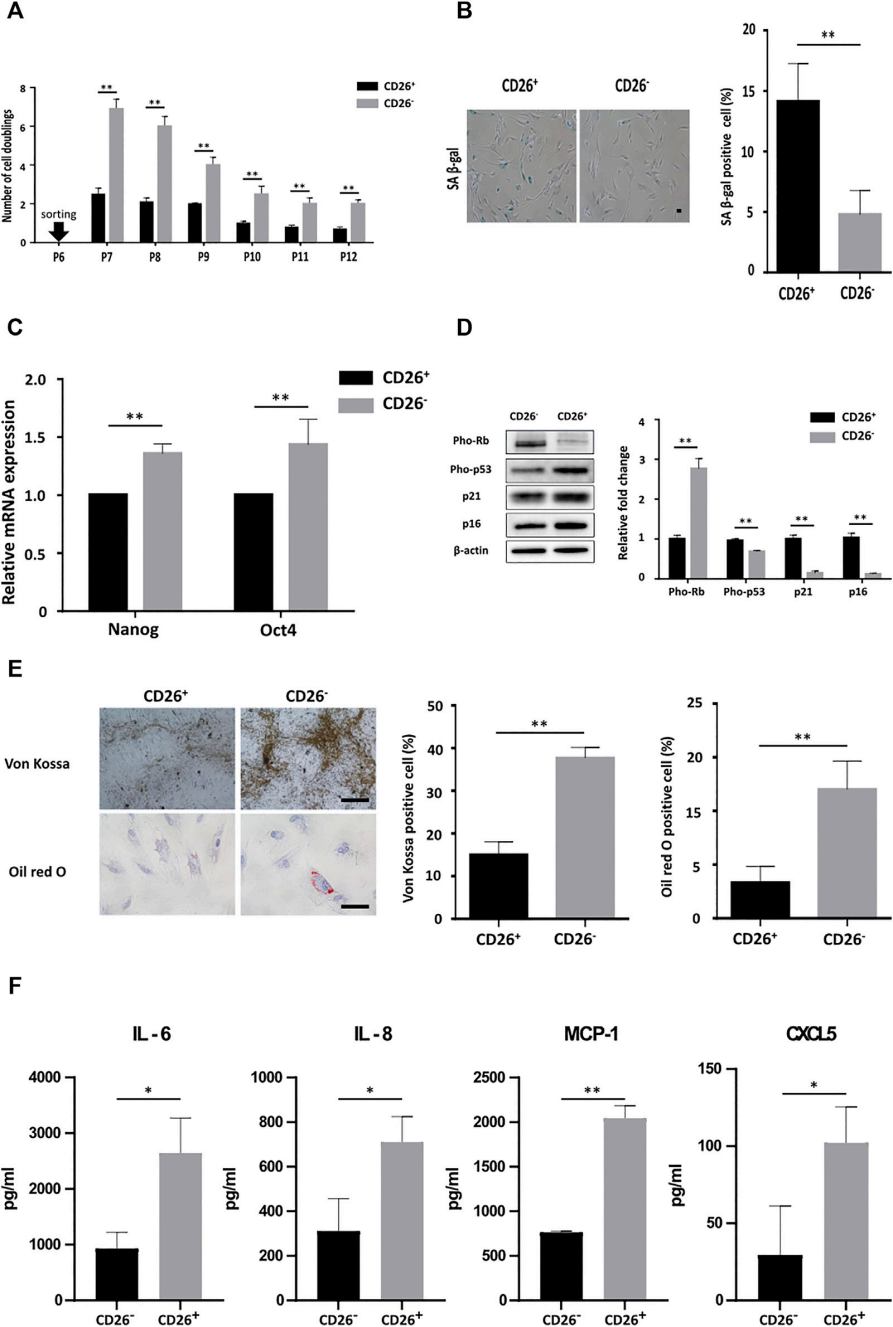
hUCB-MSCs with low CD26 expression show significantly different senescence phenotype and secretome. **(A)** Number of cell doublings of hUCB-MSCs at each indicated passage after sorting in accordance with the expression of CD26 (*n* = 3). **(B)** Representative images **(left)** and bar graph **(right)** of SA β-gal stained CD26^+^ and CD26^−^ hUCB-MSCs (*n* = 4, passage 10); Scale bar, 50 μm. **(C)** Relative mRNA expression of Oct4 and Nanog measured by RT-qPCR in CD26^+^ and CD26^−^ hUCB-MSCs (*n* = 3). **(D)** Immunoblotting images **(left)** and densitometry **(right)** of senescence-associated proteins in CD26^+^ and CD26^−^ hUCB-MSCs (*n* = 3, passage 7). (**E)** Representative images **(left)** and bar graph **(right)** of Von Kossa and Oil red O stained CD26^+^ and CD26^−^ hUCB-MSCs (*n* = 3, passage 7) Scale bar, 25 μm. **(F)** Multiplex human cytokine analysis of conditioned medium from CD26^+^ or CD26^−^ hUCB-MSCs (*n* = 3, passage 7). Abbreviation: hUCB-MSCs, human umbilical cord blood mesenchymal stem cells; SA β-gal, senescence-associated β galactosidase; RT-qPCR, quantitative reverse transcription PCR. *p < 0.05; **p < 0.01.

As shown in Figure 1D, the expression of CD26 was not uniformly elevated in all cells in late passage. Some of these cells in late passage maintained low or undetectable expression of CD26. We hypothesized that subgroups of cells showing different levels of CD26 may exhibit different characteristics in relation to senescence. We sorted cells by fluorescence-activated cell sorting (FACS) based on CD26 expression (CD26^+^/CD26^−^; Supplementary Figure S3) and cultured these cells separately. As expected, CD26^+^ cells significantly grew slower than CD26^−^ cells, and the gap of the proliferation rate between these cells was maintained across several subcultures (Figure 4A). The impairment of proliferation in CD26^+^ cells was due to accelerated cellular senescence, evidenced by the increased ratio of SA β-gal-stained cell (Figure 4B) and the diminished expression of Oct4 and Nanog (Figure 4C). Changes in the Rb and p53 phosphorylation status and the accumulation of p21 and p16 (Figure 4D) also demonstrated premature onset of senescence in the CD26^+^ cells. In addition, the multilineage differentiation potential was markedly reduced in the CD26^+^ cells, as demonstrated by their reduced staining in Von Kossa or Oil red O (Figure 4E). These results suggested that *in vitro* cultured hUCB-MSCs can exhibit varying levels of cellular senescence at the same passage, and that CD26 could be considered a selective marker of senescent cells in heterogenous populations of MSCs.

Senescence involves characteristic changes to secreted cytokine, namely a prominent increase of pro-inflammatory soluble factors, referred to as a senescence-associated secretory phenotype (SASP) (Coppé et al., 2008; Coppé et al., 2010; Freund et al., 2010). We examined whether MSCs that had been sorted based on their CD26 expression would show different SASP related cytokine secretion profile. The secreted cytokine levels in cell culture medium conditioned with CD26^+^ or CD26^−^ cells were measured by multiplex cytokine analysis. As expected, the secretion of representative SASP interleukins and chemokines such as IL-6, IL-8, MCP-1, and CXCL5 was increased in CD26^+^ hUCB-MSCs even in early-passage (Figure 4F). this finding suggested that sorting of hUCB-MSCs to select with low or negative CD26 expression would improve the cytokine secretion profiles of hUCB-MSCs.

### Sorting hUCB-MSCs by CD26 Expression Augments the Therapeutic Potential of MSCs in Mouse Emphysema Model

**FIGURE 5:**
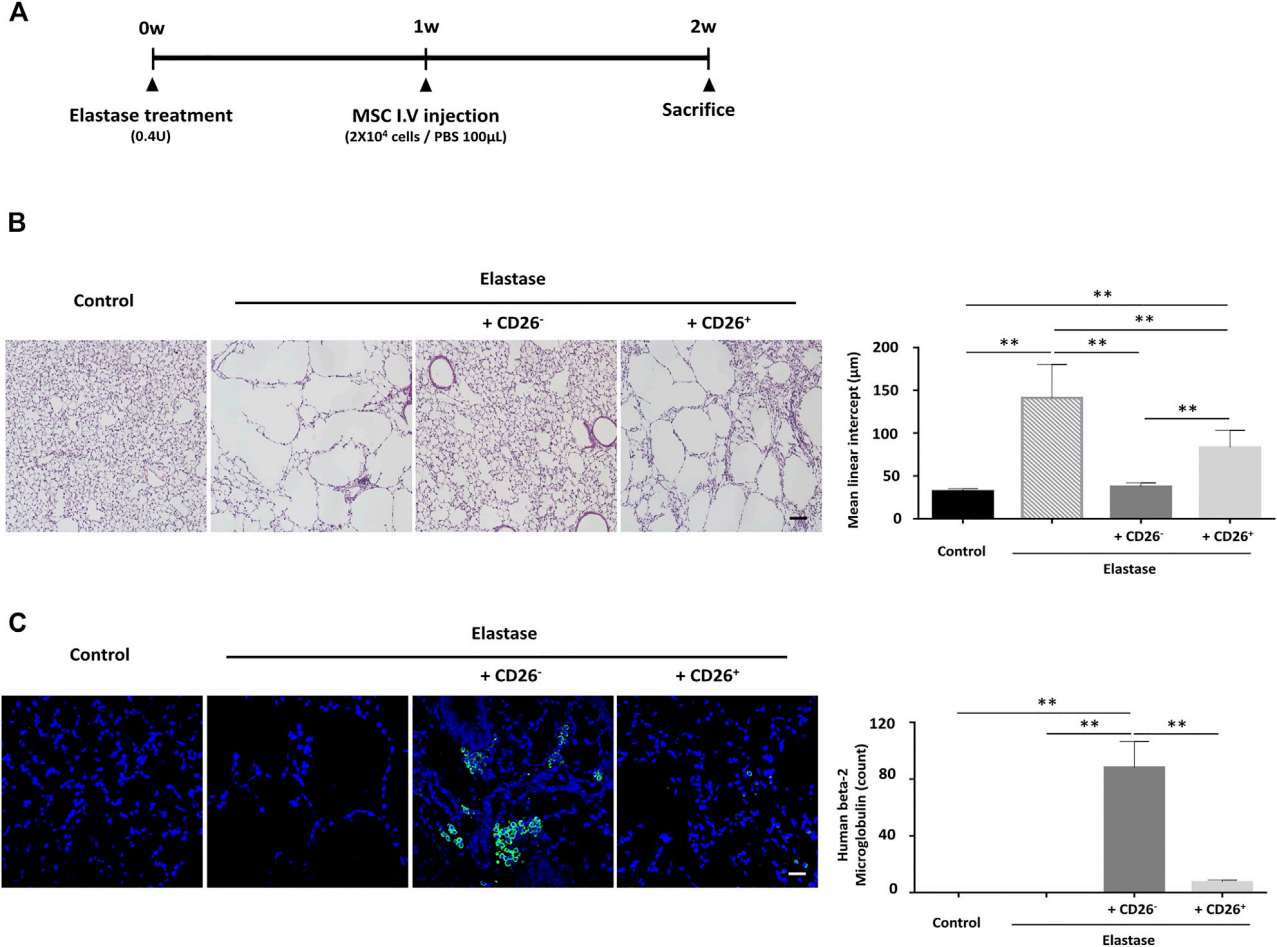
hUCB-MSCs with low CD26 expression demonstrate enhanced therapeutic effects in a mouse emphysema model. **(A)** Time schedule for the elastase-induced emphysema mouse model used in the experiment. **(B)** Representative images of H&E-stained lung tissue showing alveolar wall destruction by elastase **(left)**. Bar graph of mean linear intercept from images of H&E staining **(right)** (*n* = 12); Scale bar, 50 μm. **(C)** Representative images of mouse lung tissue immunostained with human beta-2 microglobulin (green). Nuclear DNA was counterstained with Hoechst 33342 (blue) **(left)**. Bar graph of human beta-2 microglobulin-positive cell counts **(right)** (*n* = 3); Scale bar, 50 μm. Abbreviation: H&E, Hematoxylin and Eosin. *p < 0.05; **p < 0.01.

To determine whether the therapeutic effects of hUCB-MSCs could indeed benefit from the removal of CD26^+^ cells *in vivo*, we tested these cells in an elastase-induced emphysema mouse model. The timeline of elastase treatment and IV injection of hUCB-MSCs in this model is depicted in Figure 5A. The mice were sacrificed at 1 week after the MSC injection, and the lungs were sectioned for histological analysis. We assessed the mean linear intercept as the indicator of the severity of emphysema. Elastase treatment clearly induced severe emphysema-like alveoli, resulting in increased mean linear intercept. The injection of CD26^+^ MSCs alleviated the damage to the alveolar septa to some degree. Importantly however, the injection of CD26^−^ MSCs further mitigated the destruction of the alveolar septa, and the mean linear intercept was close to the naïve group (Figure 5B). We also performed immunofluorescence analysis for human beta-2 microglobulin to assess the level of engraftment of the injected MSCs. As shown in Figure 5C, there were significantly more resident human cells in CD26^−^ hUCB-MSCs injected mice. This outcome demonstrates that administration CD26^−^ hUCB-MSCs rather than heterogeneous population could enhance the therapeutic effects of MSCs *in vivo*.

## Discussion

Here, we report on the gene expression profile of senescent hUCB-MSCs and identify CD26 as one of significantly upregulated proteins in cellular aging process. Taken together with a previous report that found CD26 to be the targetable membranous protein of senescent fibroblasts (Kim et al., 2017), Our current results suggest that the actions of CD26 about cellular senescence may be common across different cell types.

Subculturing of MSCs is essential for their clinical use as the number of cells that can be harvested from donors is insufficient for therapeutic purposes. Aging of MSCs is therefore unavoidable due to the need to expand these cells in culture. Since senescent MSCs have been reported to show poor therapeutic effects in multiple prior studies [reviewed in (Liu et al., 2020)], there have been attempts to rejuvenate or selectively eliminate aged MSCs prior to therapeutic use. We investigated whether CD26 can be used for either approach in our present analysis. Our results revealed that the suppression of CD26 expression by siRNA (Figure 2D) or CD26 activity by pharmacologic inhibitor K579 (Figure 2A) could slow down the progression of cellular aging in MSCs. Our finding that 579 alone could effectively inhibit cellular senescence is crucial as it suggests the possibility of generating enhanced MSCs without any genetic modification of CD26. Although non-integrative viral vectors such as adeno-associated virus (AAV) and nonviral vectors for nucleic acid delivery have been developed for MSCs (Gerace et al., 2017; Hamann et al., 2019), the availability of pharmacologic inhibitor will be beneficial with respect to safety and scalability in the production of MSCs-based medicinal products. Furthermore, a single treatment of K579 was found to be sufficient to maintain a superior growth rate through multiple passaging compared to naïve cells in our experiment, again illustrating the potency of CD26 inhibition in relation to the rejuvenation of MSCs.

It was intriguing that continuous subculturing led to a heterogenous mixture of hUCB-MSCs with respect to the level of CD26 expression (Figure 1E). The heterogeneity of MSCs has been well-studied at the level of donor (Choudhery et al., 2014) or extracted tissue (Vasandan et al., 2014). However, a wide distribution of CD26 expression in late passage hUCB-MSCs cannot be simply explained by the heterogeneity of the “original” hUCB-MSCs prior to division *in vitro* as early passage hUCB-MSCs showed homogenous low expression level of CD26. Moreover, any CD26^+^ hUCB-MSCs at the time of harvest would likely be counter-selected considering their slow proliferation. Hence, the different CD26 levels within hUCB-MSCs that we observed is most likely derived from the *in vitro* culturing process. There have been previous attempts to detect senescent cells from heterogenous cell mixture using the intracellular ATP level, autofluorescence (Block et al., 2017) and membranous protein such as CD264 (Madsen et al., 2017). We here demonstrated that the selection of hUCB-MSCs with low CD26 expression could improve gene expression profile related to senescence (Figure 4D), the differentiation potential of MSCs (Figure 4E), and most importantly, *in vivo* therapeutic effects (Figure 5B). This suggests the possibility of using CD26 as another possible surface cell marker to manufacture MSC therapeutics with enhanced efficacy.

SASP refers to the senescence-related changes in cytokine secretion and is observed in a number of different cell types, including liver stellate cells, endothelial cells, and fibroblasts (Shelton et al., 1999; Schnabl et al., 2003). Nevertheless, the SASP of MSCs is of particular concerns from a clinical perspective because many SASP components are pro-inflammatory cytokines that can hamper the immunomodulatory activity of MSCs. We observed significant differences of cytokine level in well-established SASP factor such as IL-6, IL-8, and MCP-1 between CD26^+^ and CD26^−^ hUCB-MSCs (Figure 4F), which corroborated the effectiveness of CD26 as functional surface marker of MSCs. Reducing SASP factors in culture medium could be advantageous as this will suppress two pathways: the activation of pro-inflammatory program (Gnani et al., 2019), and the induction of senescence process in either autocrine (Kuilman et al., 2008) or paracrine (Vassilieva et al., 2018) manner.

We also demonstrated that CD26^−^ hUCB-MSCs have a superior therapeutic efficacy in a mouse emphysema model (Figure 5B). Emphysema is the destruction of lung parenchyma found in patients of chronic pulmonary obstructive disease (COPD). COPD has been the strong candidate for MSC therapy due to lack any other treatment that can halt or reverse its progression. The first study of MSC therapy in a mouse model of elastase-induced pulmonary emphysema was performed in 2006 (Shigemura et al., 2006). Since then, multiple preclinical studies have reported the effectiveness of MSCs in chemically induced emphysema (Antunes et al., 2017). However, despite these hopeful results in animal models, clinical trials have so far failed to demonstrate significant improvement of patient outcomes, such as in pulmonary function or the mortality rate (Weiss et al., 2013). The development of MSCs with “superior” performance is therefore likely to be crucial along with a more optimized treatment plan such as the dosage and route of administration (Broekman et al., 2018). In our results, the administration of CD26^+^ MSCs produced a significant alleviation in the damage to the alveolar septa, but the use of CD26^−^ MSCs yielded even better histologic outcomes, suggesting that modification of MSCs to enhance their therapeutic efficacy can be beneficial for diseases that were already proven to be effective with naïve MSCs. It was noteworthy that a better retention of hUCB-MSCs in the mouse lung also became evident with CD26^−^ hUCB-MSCs. There has been some dispute among researchers about whether MSC could be retained over meaningful duration (Hoffman et al., 2011; Eggenhofer et al., 2012). Our results suggesting the inhibition of CD26 could improve the engraftment profile of hUCB-MSCs both *in vitro* (Figure 3A) and *in vivo* (Figure 5C) implies that the migration into lung of MSCs might be relevant with clinical outcomes, at least in a mouse emphysema model.

## Data Availability

The datasets presented in this study can be found in online repositories. The names of the repository/repositories and accession number(s) can be found below: https://www.ncbi.nlm.nih.gov/geo/, GSE183995.
