# Supplementary figures and images for: CD26 Inhibition Potentiates the Therapeutic Effects of Human Umbilical Cord Blood-Derived Mesenchymal Stem Cells by Delaying Cellular Senescence
Source: Front Cell Dev Biol. 2022 Feb 1;9:803645. doi: 10.3389/fcell.2021.803645 (PMC8846329; doi:10.3389/fcell.2021.803645)

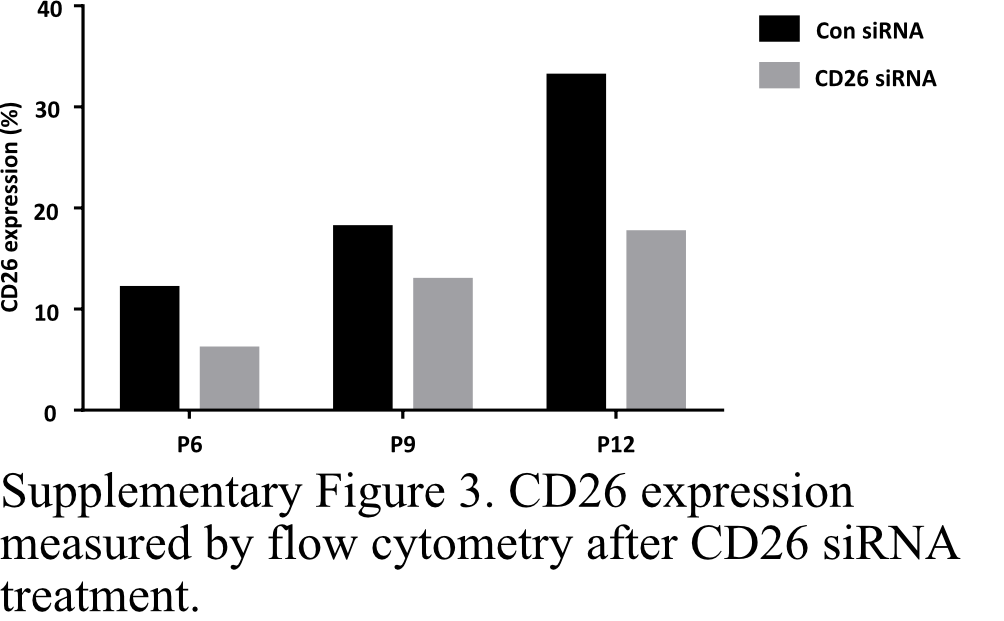

Supplement: Supplementary file 1 [file Image3.TIFF]

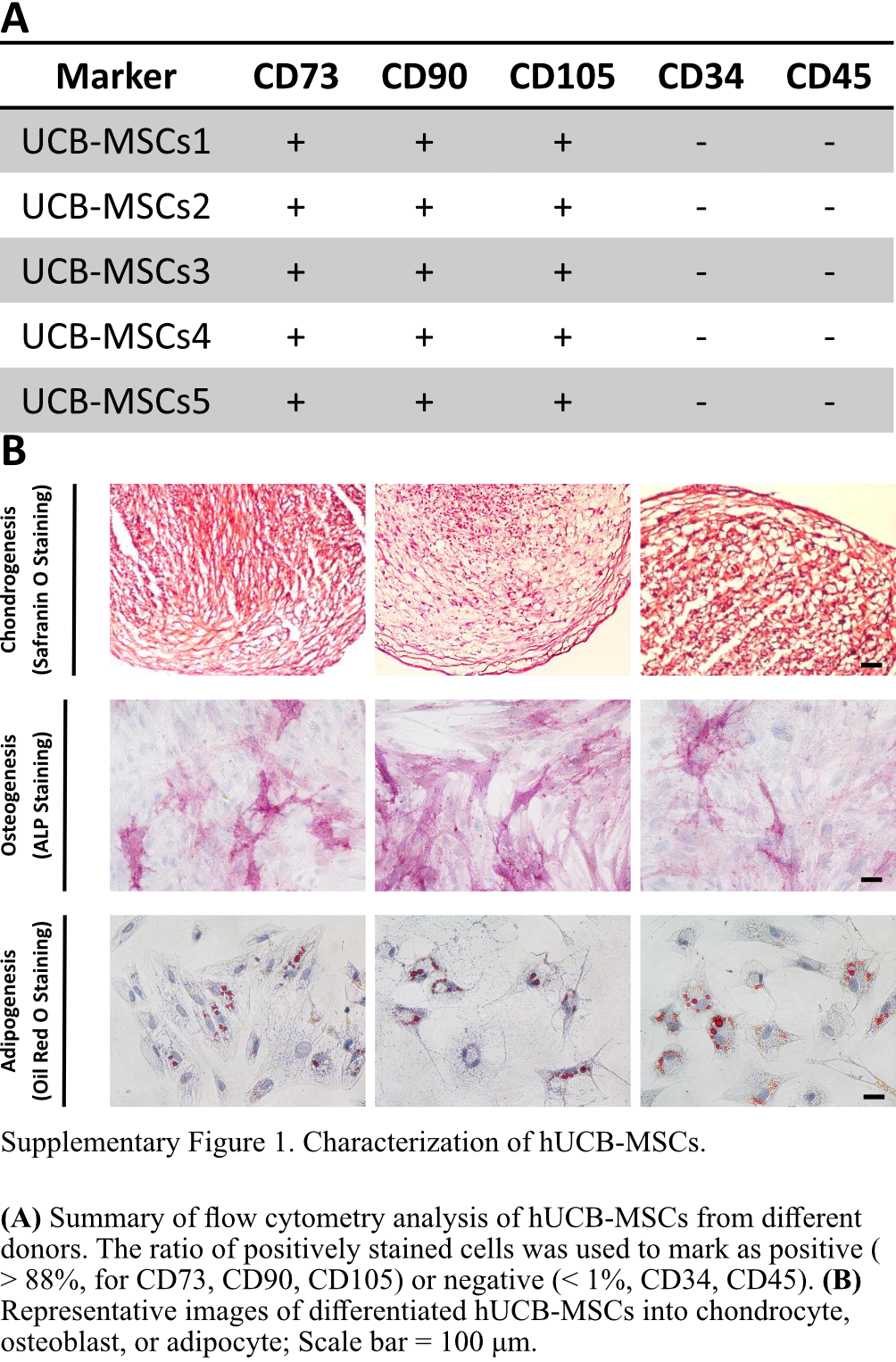

Supplement: Supplementary file 3 [file Image1.TIFF]

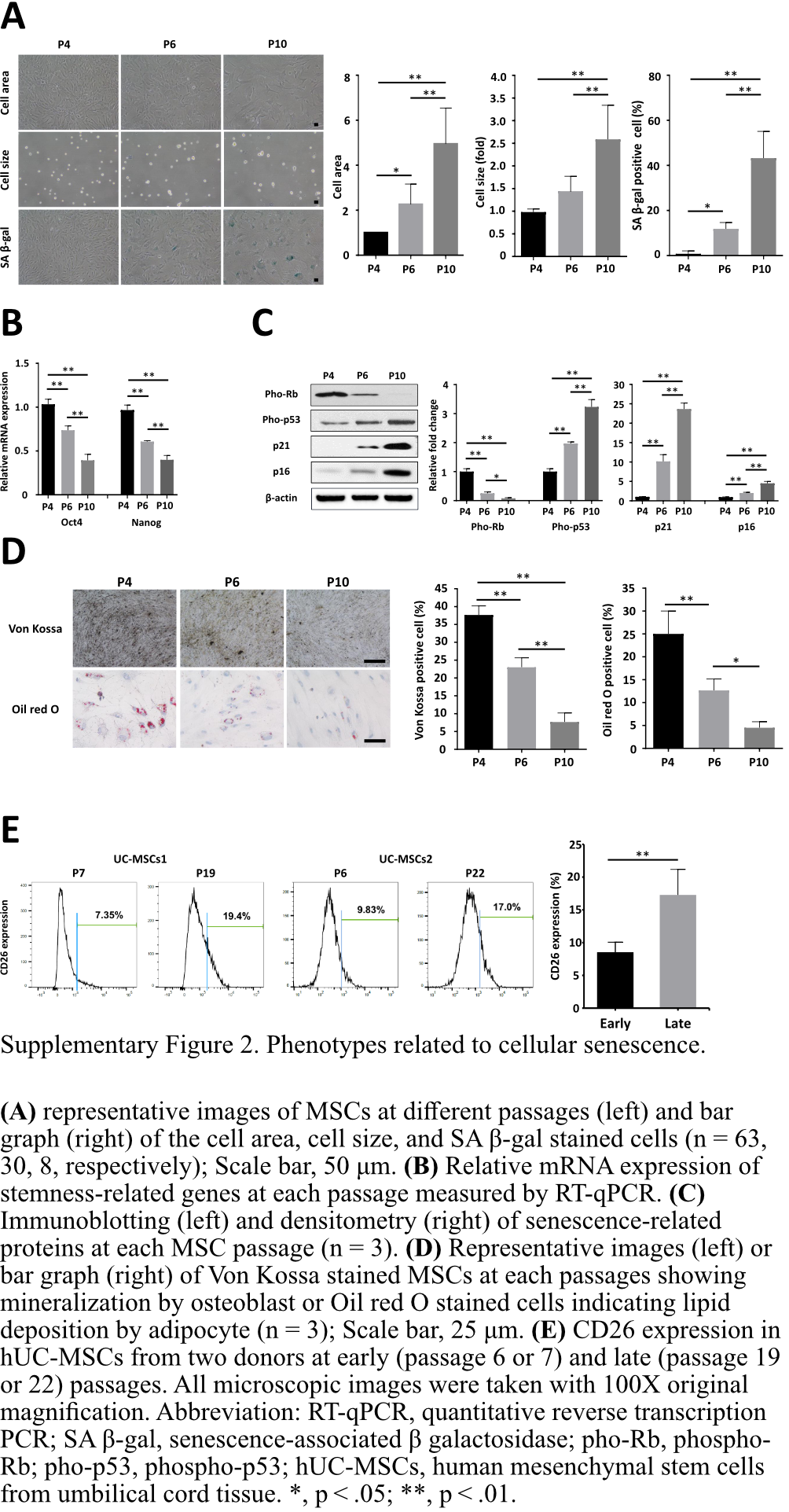

Supplement: Supplementary file 5 [file Image2.TIFF]

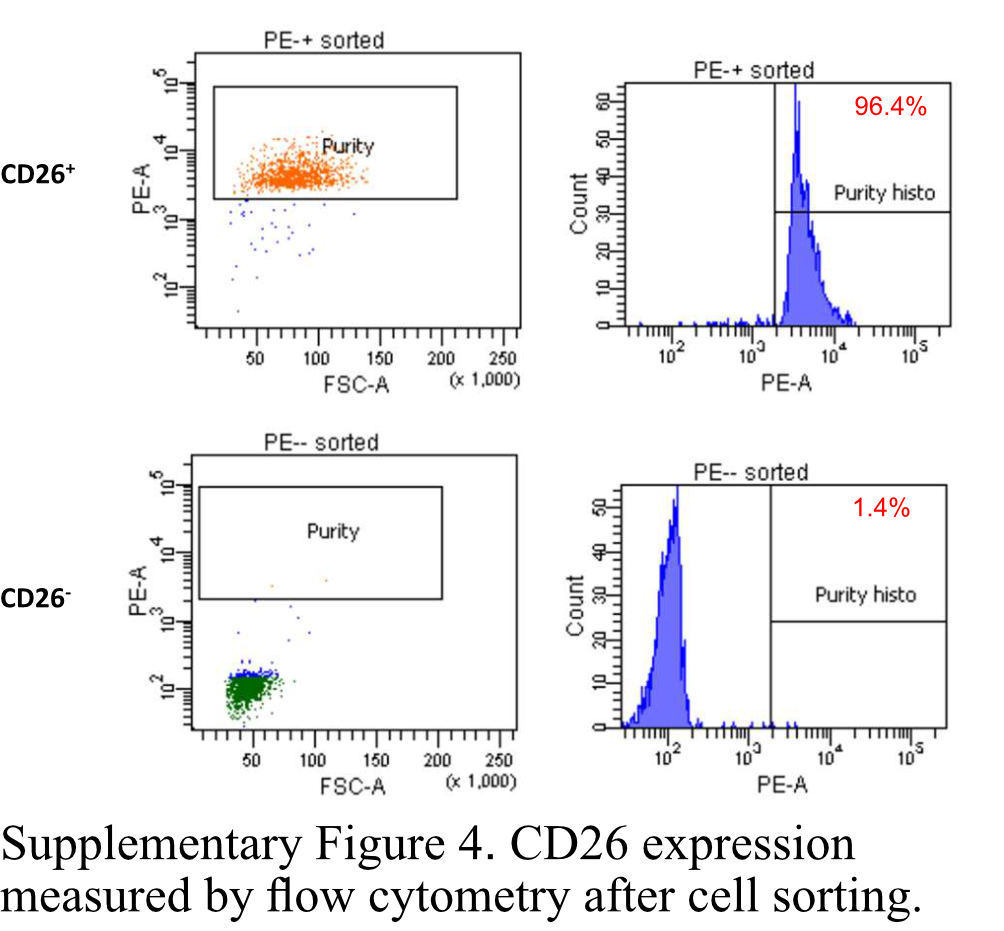

Supplement: Supplementary file 6 [file Image4.TIFF]
