# Supplementary material for: CD26 Inhibition Potentiates the Therapeutic Effects of Human Umbilical Cord Blood-Derived Mesenchymal Stem Cells by Delaying Cellular Senescence
Source: Front Cell Dev Biol. 2022 Feb 1;9:803645. doi: 10.3389/fcell.2021.803645 (PMC8846329; doi:10.3389/fcell.2021.803645)
Supplement: Supplementary file 4 [file Table1.DOCX]

Supplementary Table S1. Sequence of qPCR primers and siRNA used in the experiments

| Target gene | | Sequence (5’ -> 3’) |
| --- | --- | --- |
| Nanog | Forward | atgcctcacacggagactgt |
|  | Reverse | cagggctgtcctgaataagc |
| Oct4 | Forward | caatttgccaagctcctga |
|  | Reverse | agatggtcgtttggctgaat |
| GAPDH | Forward | agccacatcgctcagacac |
|  | Reverse | gcccaatacgaccaaatcc |
| CD26  siRNA | Ⅰ | CACUCUAACUGAUUACUUA |
|  | Ⅱ | UAGCAUAUGCCCAAUUUAA |
|  | Ⅲ | CAAGUUGAGUACCUCCUUA |
|  | Ⅳ | UAUAGUAGCUAGCUUUGAU |
| Scrambled  control  siRNA | Ⅰ | UGGUUUACAUGUCGACUAA |
|  | Ⅱ | UGGUUUACAUGUUGUGUGA |
|  | Ⅲ | UGGUUUACAUGUUUUCUGA |
|  | Ⅳ | UGGUUUACAUGUUUUCCUA |
